# Venom composition of the ant *Tetraponera rufonigra* reveals insights into the evolution of dimeric ant venom peptides

**DOI:** 10.1007/s00018-026-06293-z

**Published:** 2026-06-12

**Authors:** Suwatjanee Naephrai, Hue N. T. Tran, Thomas Durek, Axel Touchard, Vanessa Schendel, William Atherton, Irina Vetter, Panchika Prangkio, Samuel D. Robinson

**Affiliations:** 1 Department of Chemistry, Faculty of Science, Chiang Mai University, Chiang Mai, 50200 Thailand; 2 Graduate Program in Biotechnology, Multidisciplinary and Interdisciplinary School, Chiang Mai University, Chiang Mai, 50200 Thailand; 3 Institute for Molecular Bioscience, The University of Queensland, Brisbane, QLD 4072 Australia; 4 Australian Research Council Centre of Excellence for Innovations in Peptide and Protein Science, The University of Queensland, Brisbane, QLD 4072 Australia; 5 CNRS, UMR Ecologie des forêts de Guyane – EcoFoG, Campus Agronomique, BP 316, Kourou Cedex, 97379 France; 6 School of Pharmacy and Pharmaceutical Sciences, The University of Queensland, Brisbane, QLD 4102 Australia; 7 Center of Excellence in Material Science and Technology, Faculty of Science, Chiang Mai University, Chiang Mai, 50200 Thailand

**Keywords:** Formicidae, Pseudomyrmecinae, Sting, Pain, Homodimer, Heterodimer, Convergent evolution

## Abstract

The majority of ant species are venomous, and in most lineages, peptide toxins dominate venom composition. Here we investigated the venom of the ant *Tetraponera rufonigra* (subfamily Pseudomyrmecinae). The major active component, Tr1a, is a homodimeric peptide that is cytolytic, activates mammalian sensory neurons and is insecticidal. Structural analyses show that Tr1a consists of two identical peptide chains arranged as antiparallel α-helices and covalently linked by three interchain disulfide bonds, forming a symmetrical amphipathic dimer. We demonstrate that dimeric venom peptides with similar biological activities have evolved independently multiple times across ants. The repeated evolution of dimeric venom peptides in ants highlights the evolutionary plasticity of ant venom and the role of convergence in shaping ant venom composition.

**Supplementary Information:**

The online version contains supplementary material available at 10.1007/s00018-026-06293-z.

## Introduction

Ants (Formicidae) are eusocial insects of the order Hymenoptera. The majority of ant species are venomous, and venom typically plays an important role in both predation and defense. With few exceptions, the major active components of stinging ant venoms are peptides [1–3].

The ant subfamily Pseudomyrmecinae is a lineage of arboreal ants comprising three genera: *Pseudomyrmex* (155 described species, distributed in the Neotropics), *Myrcidris* (monotypic, Neotropics), and *Tetraponera* (87 species, Afrotropics, Indomalaya and Australasia) [4]. Venom composition has been partially characterized in several *Pseudomyrmex* species [5–8] whereas knowledge of *Tetraponera* venoms is more limited [9]. Available data indicate that the venoms of pseudomyrmecine ants are superficially similar to those of other early-diverging ant lineages, including the Myrmeciinae and certain ponerine ants. For each of these lineages, cytolytic amphipathic peptides, including some dimeric peptides, are among the dominant venom components [10, 11, 9, 7]. These similarities are suggestive of conserved venom peptide scaffolds and activities among these early-diverging ant lineages. Expanding taxonomic sampling of ant venoms is important both for discovering new bioactive peptides and for improving our understanding of ant venom evolution. In particular, characterizing venoms of understudied pseudomyrmecine species may help clarify the origins and diversification of peptide toxins within ants.

**Fig. 1:**
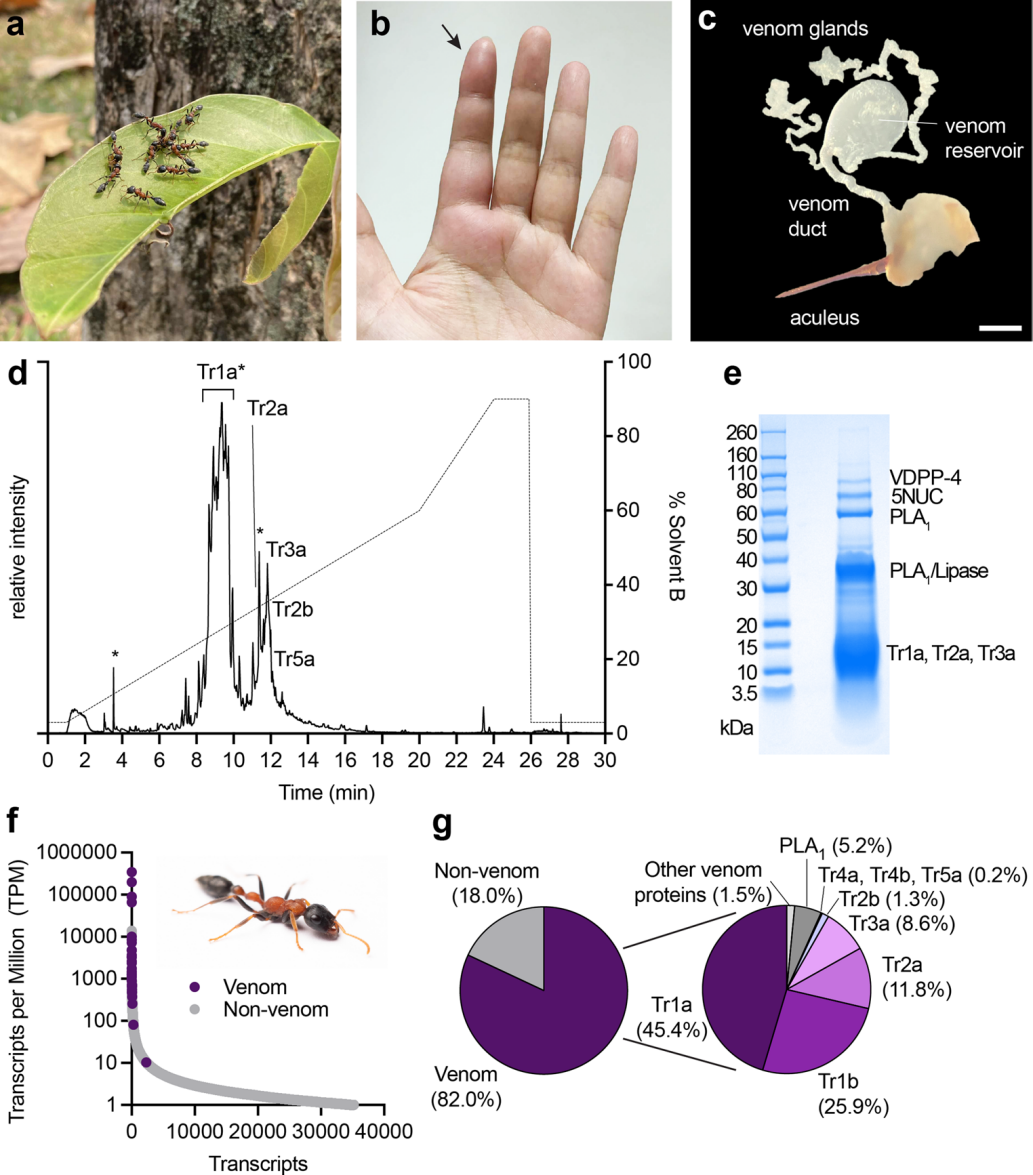
The venom composition of *T. rufonigra*. **A** Adult workers of *T. rufonigra* (~ 10–12 mm in length). **B** Human hand (S.N.) ~ 1 h after accidental envenomation by *T. rufonigra*, illustrating local swelling and redness. Arrow indicates site of envenomation. **C** Dissected venom apparatus of *T. rufonigra*. Scale bar ≈ 1 mm. **D** Total ion chromatogram from LC-MS/MS analysis of reduced and alkylated *T. rufonigra* venom with labelled peaks corresponding to identified venom peptides (venom peptide sequences are shown in Table 1). Asterisks mark derivatives of identified venom peptides. **E** SDS-PAGE gel of whole *T. rufonigra* venom (10 µg) labelled with major venom proteins identified by LC-MS/MS. **F** Venom component-encoding transcripts (i.e., those encoding peptides and proteins detected in the venom itself; highlighted in purple) constitute almost all the most highly expressed transcripts. Inset: *T. rufonigra* adult female worker (~ 11 mm in length). **G** Venom component-encoding transcripts comprised 82.0% of total venom gland transcriptome expression. Of these, transcripts encoding venom peptides and venom proteins constituted 93.3% and 6.7%, respectively

In this study, we investigated the venom of the arboreal bicoloured ant, *Tetraponera rufonigra* (Pseudomyrmecinae) from tropical south-east Asia (Fig. 1a). Envenomation by *T. rufonigra* causes localized pain and inflammation in humans (Fig. 1b). We found that the primary active component in the venom, Tr1a, is a homodimeric amphipathic peptide with cytolytic activity. Although Tr1a shares structural and functional features with dimeric peptides previously described from *Pseudomyrmex* and other ant venoms, our analyses indicate that it evolved independently. This study highlights the evolutionary plasticity of ant venoms and the important role of convergence in shaping ant venom composition.

## Materials and methods

### Venom and venom apparatus collection

Adult female worker caste *T. rufonigra* (127 individuals) were collected from a single colony at the Chiang Mai University campus, Chiang Mai, Thailand. No envenomation experiments were performed. Envenomation of researchers (S.N. and S.R.) occurred during collection of the ants for this study.

Venom was squeezed from the dissected venom reservoirs and venom ducts of 127 individuals into 20 µL water and lyophilized. Dried venom was reconstituted in 100 µL of Milli-Q water, and the total amount of venom was estimated from the A_280_ measurement using a Nanodrop spectrophotometer (Thermo Fisher, Waltham, MA, USA). The venom apparatus used for RNA-seq (25 individuals) was placed directly in RNAlater (Invitrogen).

### Transcriptome sequencing and assembly

Total RNA was extracted from the venom apparatus of *T. rufonigra* using TRIzol (Life Technologies, Carlsbad, CA, USA). Library preparation was performed at the University of Queensland Sequencing Facility (University of Queensland, Brisbane, Australia), and sequencing was conducted in conjunction with services provided by the Australian Genome Research Facility (Australia). RNA-Seq libraries (in a batch of 14 samples) were generated using the Illumina Stranded mRNA Library Ligation kit and Illumina RNA UD Indexes Ligation according to the standard manufacturer’s protocol. 1–80 ng of total RNA was enriched for mRNA using an oligo-dT bead isolation method, and the mRNA was fragmented in a heat fragmentation step. cDNA was synthesized from the fragmented RNA using random primers. The first strand cDNA was converted into dsDNA in the presence of dUTP to prevent subsequent amplification of the second strand and thus maintain the strand orientation of the original mRNA. The 3’ ends of the cDNA were adenylated and pre-index anchors were ligated. The libraries were amplified with either 13 cycles (80 ng input RNA) or 16 cycles (1–12 ng input RNA) of PCR, incorporating unique indexes for each sample to produce libraries ready for sequencing. The libraries were quantified on the Revvity LabChip GX Touch with the DNA High Sensitivity Reagent kit and pooled in equimolar ratios.

Sequencing was performed using the Illumina NovaSeq X Series sequencer. The library pool was diluted and denatured according to the standard NovaSeq X protocol and sequenced to generate paired-end 151 bp reads using a NovaSeq X Series 10B Reagent Kit (300 cycles). After sequencing, fastq files were generated using bcl2fastq2 (v2.20.0.422), and sequencing adapters were trimmed. The first cycle of each insert read was also trimmed due to expected low diversity.

Additional adapter trimming of demultiplexed raw reads was performed using fqtrim v0.9.7 [12], followed by quality trimming and filtering using prinseq-lite v0.20.4 [13]. Error correction was performed using BBnorm tadpole, part of the BBtools package. Trimmed and error-corrected reads were assembled using Trinity v2.15.1 [14] with a k-mer length of 31 and a minimum k-mer coverage of 2. Assembled transcripts were annotated using a BLASTX [15] search (E value setting of 1e^− 3^) against the UniRef90 database. Estimates of transcript abundance (transcripts per million [TPM]) were performed using the RSEM [16] plugin of Trinity (align_and_estimate_abundance). Using TransDecoder v5.5.0, transcripts were translated and filtered to identify open-reading frames (greater than 30 amino acid residues). This was used as a search database for ProteinPilot.

### Gel electrophoresis

*T. rufonigra* venom (10 µg) was denatured (70 °C, 10 min) in Bolt LDS Sample Buffer (Invitrogen) and separated on a 4–12% Bis-Tris Plus Mini Protein Gel (Invitrogen) (150 V, 28 min). Protein molecular weights were estimated by comparison with the Novex Sharp Pre-stained Protein Standard (Invitrogen). The gel was stained with SimplyBlue™ SafeStain (Invitrogen) for 1 h and then destained overnight in Milli-Q water. Five protein bands were excised and reduced and alkylated, in gel, according to the protocol described by Hale et al. [17]. Excised bands were incubated for 90 min at 37 °C in reduction/alkylation reagent (50% (v/v) ammonium carbonate, 48.75% acetonitrile (ACN), 1% 2-iodoethanol, 0.25% triethylphosphine), rinsed with water then dehydrated with 100% ACN. Trypsin (20 ng/L), prepared according to the manufacturer’s instructions (Sigma-Aldrich, St. Louis, MO), was added to the gel pieces and then incubated for 16 h at 37 °C. Reactions were quenched with formic acid (final concentration of 1%) and the samples were analyzed by LC-MS/MS.

### Mass spectrometry

A combination of top-down and bottom-up proteomics of native, reduced and alkylated, and reduced, alkylated and trypsin-digested venom was used to examine the polypeptide composition of *T. rufonigra* venom. Aliquots of venom (10 µg each) were dried by vacuum centrifugation. Gas phase reduction and alkylation was performed according to the protocol described by Hale et al. [17] 100 µL of reduction/alkylation reagent (50% (v/v) ammonium carbonate, 48.75% acetonitrile (ACN), 1% 2-iodoethanol, 0.25% triethylphosphine) was added to the lid of each 1.5 mL tube containing dried venom, which was then inverted, closed, and incubated at 37 °C for 90 min. One aliquot of reduced and alkylated venom was then digested by incubating with trypsin (20 ng/µL) overnight at 37 °C, according to the manufacturer’s instructions (Sigma-Aldrich).

Three venom samples (10 µg each) – native venom; reduced and alkylated venom; and reduced, alkylated and trypsin-digested venom – were analyzed on a ZenoTOF 7600 (SCIEX) mass spectrometer coupled to a nanoAcquity UPLC system (Waters). Approximately 1 µg of each sample was injected onto a Waters nanoEase M/Z HSS T3 C18 column (300 μm × 150 mm, 1.8 μm, 100 Å). The mobile phases were A: 0.1% formic acid in water and B: 0.1% formic acid in ACN. Peptides were loaded in 3% B. The LC was held at 3% B for 60 s, and peptides were eluted with a gradient of 3–60% B over 20 min, 60–90% over 4 min at a flow rate of 7 µL/min. MS1 spectra were acquired with mass range, 300–2200 *m*/*z*; accumulation time, 0.1 s. The 10 most intense monoisotopic precursors with intensity > 100 cps, with charge states 2–6 were selected for MS2 fragmentation by CID: TOF MSMS mass range, 100–2000 *m*/*z*; accumulation time, 0.1 s. Using ProteinPilot v5.0 (SCIEX), MS/MS spectra were searched against the translated venom-apparatus transcriptome (MS and MS/MS tolerance of 0.05 and 0.1 Da, respectively). False discovery rate analyses were generated by ProteinPilot default method, which uses a decoy database.

Transcripts encoding venom components were then manually examined using the Map-to-Reference tool of Geneious v10.2.6 [18], where “masked” homologues were identified, contaminant transcripts (derived from multiplexing) and erroneous transcripts (derived from misassembly) were discarded. This information was then reincorporated back into the transcriptome, estimation of transcript abundance repeated, and a second, final ProteinPilot search performed. Peptides identified by ProteinPilot were validated by comparison of experimentally derived MS/MS peaks against a theoretical peak list generated using MS-Product in ProteinProspector v6.8.1 (https://prospector.ucsf.edu/prospector/cgi-bin/msform.cgi?form=msproduct).

To investigate the glycosylation site of the toxin Tr2a, native venom was subjected to LC-MS/MS analysis using electron-activated dissociation (EAD) on the ZenoTOF 7600: charge states 2–6 were selected for MS2 fragmentation, TOF MSMS mass range: 100–1500 *m*/*z*; accumulation time, 0.055 s. Spectra were searched using PEAKS Studio (SCIEX) against the translated *T. rufonigra* venom gland transcriptome. Search parameters included a 0.05 Da precursor mass tolerance and a 0.1 Da fragment mass tolerance.

### Calcium imaging of F11 cells

F11 cells (mouse neuroblastoma $\:\times\:$ DRG neuron hybrid) were maintained on Ham’s F12 media supplemented with 10% FBS, 100 µM hypoxanthine, 0.4 µM aminopterin, and 16 µM thymidine (Hybri-Max, Sigma Aldrich). 384-well imaging plates (Corning, Lowell, MA, USA) were seeded 24 h prior to calcium imaging, resulting in ~ 90% confluence at the time of imaging. Cells were loaded for 30 min at 37 °C with Calcium 4 assay component A in physiological salt solution (PSS; 140 mM NaCl, 11.5 mM D-glucose, 5.9 mM KCl, 1.4 mM MgCl_2_, 1.2 mM NaH_2_PO_4_, 5 mM NaHCO_3_, 1.8 mM CaCl_2_, 10 mM HEPES) according to the manufacturer’s instructions (Molecular Devices, Sunnyvale, CA). Ca^2+^ responses were measured using a FLIPR^TETRA^ fluorescent plate reader equipped with a CCD camera (Ex: 470 to 490 nm, Em: 515 to 575 nM) (Molecular Devices, Sunnyvale, CA). Signals were read every second for 10 s before, and 300 s after, the addition of venom or venom fractions (in PSS supplemented with 0.1% BSA).

### Fractionation of venom by reversed-phase high performance liquid chromatography (RP-HPLC)

Crude *T. rufonigra* venom (75 µg) was separated using a Gemini NX-C18 column (250 × 4.6 mm; particle size, 3 μm; pore size, 100 Å) with a gradient of 5–60% solvent B (0.05% TFA, 90% ACN) over 55 min at a flow rate of 0.8 mL/min. Fractions were collected based on absorbance at 214 nm and then dried by vacuum centrifugation. Dried fractions were resuspended in 20 µL of pure water and stored at − 20 °C.

### Peptide synthesis

Tr1a, Tr1a[Met25Nle] and Tr3a were produced using 9-Fluorenylmethoxycarbonyl (Fmoc) solid-phase peptide synthesis at 0.1 mmol scale. Protecting groups used were Cys/Gln(Trt), Ser/Thr(tBu), Asp(OMpe), Arg(Pbf), and Lys/His/Trp(Boc). Peptides were assembled on ProTide Rink amide resin (loading 0.2 mmol/g, CEM Corp, Matthews, NC, USA) on a CEM Liberty Prime HT24 microwave synthesizer (CEM Corp, Matthews, NC, USA) using *N*,*N*’-diisopropylcarbodiimide (DIC)/oxyma and Fmoc groups were removed with 25% pyrrolidine, as per manufacturer’s protocols. Peptides were released from resin by treatment with 92.5% trifluoroacetic acid (TFA)/2.5% H_2_O/2.5% trisiopropylsilane (TIPS)/2.5% 2,2′-(ethylenedioxy)diethanethiol. TFA was removed under N_2_ flow. Peptides were precipitated with 15 mL ice-cold ether, redissolved in 50% aqueous acetonitrile (ACN) and lyophilized prior to purification. Peptides were purified on a Shimadzu Prominence LC-20AT RP-HPLC system equipped with a SPD-20AV UV detector using a Agilent Eclipse XDB-C18 column (21.2 × 250 mm; particle size, 7 μm; pore size, 300 Å) using a flow rate of 20 mL/min and a gradient of 20–50% solvent B over 60 min (A: 0.05% TFA, B: 90% ACN, 0.045% TFA). Fractions were lyophilized and purity assessed using LC-MS (API 2000 paired with Agilent model 1100) and analytical RP-HPLC (ThermoFisher Hypersil Gold-C18 column, 2.1 × 100 mm; particle size, 3 μm; pore size, 175 Å; flow rate 0.5 mL/min). Pure fractions of each peptide were pooled, lyophilized and stored at room temperature until use. Stock solutions of each peptide were prepared by dissolving in 100% H_2_O (1 mM final concentration) and stored at − 20 °C.

To form the homodimers of Tr1a and Tr1a[Met25Nle], purified monomeric peptides were first dissolved in a minimal amount of 50% aqueous ACN and then diluted in 0.1 M NH_4_HCO_3_ oxidising buffer to a final concentration of 0.25 mg/mL, and stirred for 4 d at room temperature. The reaction was monitored by analytical HPLC before quenching with 0.1% TFA and purification by preparative HPLC using a flow rate of 20 mL/min and a gradient of 30–50% solvent B over 40 min.

### Peptide co-elution

An aliquot of the fraction containing Tr1a (F14) was analysed by RP-HPLC (ThermoFisher Hypersil Gold-C18 column, 2.1 × 100 mm; particle size, 3 μm; pore size, 175 Å) using a flow rate of 0.3 mL/min and a gradient of 0–60% solvent B over 30 min (A: 0.05% TFA, B: 90% ACN, 0.045% TFA). Absorbance at 214 nm was monitored. Synthetic Tr1a (0.1 nmol) and synthetic Tr1a (0.1 nmol) and Tr1a (F14) were subsequently analysed under the same conditions.

### NMR Spectroscopy

Tr1a and Tr1a[Met25Nle] was dissolved in 90% H_2_O/10% D_2_O at a concentration of approximately 1 and 5 mg/mL, respectively. One-dimensional ^1^H NMR spectra were acquired for both peptides on a Bruker Avance III 600 spectrometer equipped with a cryoprobe (Bruker GmbH, Karlsruhe, Germany) at 298 K. Spectra were referenced to water at 4.77 ppm and assigned using CCPNMR Analysis 3.3.2.1. Two-dimensional TOCSY, NOESY and ^1^-^15^ N-HSQC spectra were acquired for Tr1a[Met25Nle] under the same conditions.

Structure calculations of Tr1a[Met25Nle] were performed using Cyana 3.98.15 using experimentally-determined distance restraints obtained from 2D NOESY spectra. 100 structures of each of the 11 possible disulfide isomers were calculated and the top 20 models selected for further analysis. All models were analysed by Molprobity [19] and the results averaged for the 20 models of each disulfide isomer.

### Calcium imaging of mouse dorsal root ganglion (DRG) cells

DRG cells were isolated from 6–8-week-old male C57BL/6 mice purchased from the Animal Resources Centre. DRGs were dissociated, then cells plated in Dulbecco’s Modified Eagle’s Medium (DMEM; Gibco, MD, USA) containing 10% fetal bovine serum (FBS) (Assaymatrix, VIC, Australia) and penicillin/streptomycin (Gibco) on a 96-well poly-D-lysine-coated culture plate (Corning, ME, USA) and maintained overnight. Cells were loaded with Fluo-4 AM calcium indicator, according to the manufacturer’s instructions (ThermoFisher Scientific, MA, USA). After loading (1 h), the dye-containing solution was replaced with assay solution (Hanks’ balanced salt solution, 20 mM HEPES). Images were acquired at 10⋅ objective at 1 frame/s (excitation 485 nm, emission 521 nm). Fluorescence corresponding to [Ca^2+^]_*i*_ of ~ 250 cells per experiment was monitored in parallel using a Nikon Ti-E deconvolution inverted microscope, equipped with a Lumencor Spectra LED Lightsource. Baseline fluorescence was monitored for 30 s. At 30 s, assay solution was replaced with either assay solution (negative control), then at 1 min with test peptide (in assay solution) and monitored for 2 min before being replaced with KCl (30 mM in assay solution; positive control). Experiments involving use of mouse tissue were approved by the UQ AEC (approval 2021/AE000812) and conducted in accordance with the Australian Code of Practice for the Care and Use of Animals for Scientific Purposes, 8th edition (2013).

### Liposome leakage assay

Phospholipids used for liposome formation were purchased from Avanti Polar Lipids, Inc. A lipid mixture of 1-palmitoyl-2-oleoyl-glycero-3-phosphocholine (POPC) and 1-palmitoyl-2-oleoyl-glycero-3-phosphoethanolamine POPE in a molar ratio of 1:1, 20 mg/mL each in chloroform, was added to a round-bottom flask, followed by rotary evaporation (at 160 rpm, 90 min). The dried lipid film was rehydrated in 1 mL of 20 mM HEPES, pH 7.4, containing 80 mM 5,6-carboxyfluorescein (CF) (Sigma-Aldrich), incubated at 37 °C for 2 h and vortexed every 1 h. The resulting lipid vesicles were subjected to 15-freeze-thaw cycles, and extruded 21 times through 100-nm polycarbonate membrane (Avanti Polar Lipids). To remove non-encapsulated free CF from the liposomes, a size-exclusion PD-10 column (GE Healthcare) was employed, eluting with 20 mM HEPES pH 7.4. Liposome concentration was determined using a phosphorus assay [20]. To assess membrane permeability of peptides of interest, Tr1a and Tr3a diluted in PBS (pH 7.4) were added to CF-encapsulated liposomes in 96-well plates (100 µL in each well), using 60 µM phosopholipids. Peptide-induced liposome vesicle leakage was measured by fluorescence intensity at Ex/Em: 492/517 nm using kinetics mode every 20 s for 30 min, followed by addition of 1% Triton X-100 (positive control), using SpectraMax i3x Multi-Mode Microplate Reader (Molecular Devices, LLC, San Jose, CA, USA).

The leakage percentage was calculated with the following equation [21]:

$$\%\mathrm{Leakage}(t)=100 \times [I(t)-I_{(0)}]/[I_{(Max)} - I_{(0)}]$$

where *I*(*t*) was the fluorescence intensity at time t, *I*_(0)_ was the initial fluorescence intensity of each group, and *I*_(*Max*)_ was the maximum fluorescence intensity after addition of Triton X-100.

### Blowfly assay

Dose-response assays were conducted to evaluate the paralytic and lethal effects of Tr1a and Tr3a on adult blowflies (*Lucilia cuprina*). Flies were injected with 1 µL of peptide solution at different concentrations, and paralysis was assessed at 1 h and 24 h, with mortality recorded at 24 h post-injection. Each dose and its corresponding control were tested on ten individuals with three independent replicates.

### Structure prediction

The structure of the Tr1a was predicted using Alphafold3 [22]. Input data were two copies of the Tr1a monomer sequence (without *C*-terminal amidation). Cartoon with ball and chain representation and molecular surface representation coloured according to residue hydrophobicity were made using Alphafold3 Structure Tools.

### Circular dichroism spectroscopy

Far UV CD spectra were recorded for Tr1a[Met25Nle] (19 µM) in 10 mM K_2_HPO_4_, pH 7.4, using a Jasco J-810 CD spectropolarimeter (Easton, MD). Spectra were acquired from 260 to 185 nm, using a 1 mm path-length quartz cuvette, 1 nm step size, and averaging of 3 spectra.

### Phylogenetic reconstruction

Precursor nucleotide sequence alignments were made using the L-INS-i algorithm of MAFFT v7.490 [23]. The most appropriate evolutionary models were selected using ModelFinder [24] before reconstructing molecular phylogenies with IQ-TREE v2.2.0 [25] by maximum likelihood, and estimating branch support values using ultrafast bootstrap with 1,000 replicates. Trees were visualised in Archaeopteryx v0.9921.

### Genome analysis

Genome assemblies of *T. rufonigra* (GenBank: GCA_048542885.1) *Tetramorium bicarinatum* (GenBank: GCA_928718305.1), *Odontomachus monticola* (Genbank: GCA_048544815.1), *Myrmecia pilosula* (Genbank: GCA_048542125.1) and *Pseudomyrmex spinicola* (Genbank: GCA_048543035.1) were downloaded from NCBI Genbank. Precursor sequences of venom peptides were used as queries to search, using blastn, each genome assembly for related venom peptide genes. Identified genes were manually checked for intron/exon boundaries in Geneious v10.2.6. Mauve [26] was used to align and visualise ~ 1 Mb extractions containing the genes of interest.

### Statistical analyses

Data were plotted and analyzed using Prism v10.3.1 (GraphPad Software, San Diego, CA, USA). All data are presented as mean ± SEM.

## Results

We used a combined transcriptomic and mass spectrometry (MS)-based strategy to investigate the venom composition of *T. rufonigra*. RNA extracted from venom apparatus (venom glands, venom reservoir, venom duct, and Dufour’s gland; Fig. 1c) of 25 ants was used to generate a venom apparatus transcriptome. We obtained 231,769,688 demultiplexed paired-end reads from Illumina NextSeq RNA sequencing, which, following adaptor trimming, quality trimming and filtering and error correction, were assembled *de novo* using Trinity to yield a total of 61,648 contigs. We collected *T. rufonigra* venom by dissecting out venom reservoirs from 127 ants, squeezing the contents into water and pooling. Liquid chromatography-tandem MS (LC-MS/MS) data from three venom samples (native; reduced and alkylated; reduced, alkylated, and trypsin-digested) were searched against a database comprising the translated venom-apparatus transcriptome.

The major peaks in the total ion chromatogram of the reduced and alkylated venom corresponded to five peptides which we named (Pseudomyrmecitoxin_1_-(PSDTX_1_)-Tr1a, PSDTX_1_-Tr2a, PSDTX_1_-Tr2b, PSDTX_1_-Tr3a and PSDTX_1_-Tr5a (Fig. 1d; Table 1). Tr1a was present in the native venom sample as a homodimer (theoretical dimer mass = 5382.67, observed mass = 5382.74 Da) (Fig. S1). Tr2a was detected with a mass indicative of *N*-acetylhexosamine (HexNAc) glycosylation (theoretical mass = 4411.58 Da, observed mass = 4614.67 Da, Δmass = + 203.09) (Fig. S2a). MS/MS data were consistent with the presence of HexNAc, with characteristic oxonium ions detected at m/z 126.05 Da, 138.05 Da, 144.07 Da, 168.07 Da, 186.08 Da, and 204.09 Da (Fig. S2b). The oxonium ion ratio of 0.61, was indicative of *N*-acetylgalactosamine (GalNAc) [27]. Reanalysis of the native venom sample under electron activated dissociation (EAD) mode, suggested S34 was the likely glycosylation site of Tr3a. One additional peptide, PSDTX_1_-Tr4a/b (encoded by two distinct transcripts) was detected only in the native venom sample. Sequence PSDTX_1_-Tr1b was not detected in our LC-MS data but had high-sequence similarity and a similarly high expression level estimate to Tr1a, so was included as a putative venom component.

Examination of *T. rufonigra* venom by SDS-PAGE (Fig. 1e) revealed four major bands > 20 kDa. These were excised and analyzed by bottom-up sequencing. Bands corresponded to a dipeptidyl peptidase 4 (DPP-4), a nucleotidase, a phospholipase-A1 (PLA_1_), and lipase. Three of the venom peptides (Tr1a, Tr2a and Tr3a) were detected in the prominent band at mass < 15 kDa. Each of these proteins as well as venom allergen 3 and phospholipase A_2_ (PLA_2_) were also detected in the reduced, alkylated and trypsin-digested whole venom sample (Table S1).

Transcripts encoding the 8 venom peptides accounted for 93.3% of the venom component-encoding reads in the venom apparatus transcriptome (Fig. 1f, g). Of these, the transcripts encoding Tr1a and Tr1b were the most highly expressed, accounting for more than half of the total venom-apparatus transcriptome (542,884 TPM). Tr2a and Tr2b (99,738 TPM), and Tr3a (65,399 TPM) were also highly expressed. Venom proteins accounted for 6.7% of venom component-encoding reads in the venom apparatus transcriptome, and of these, PLA_1_ was the most highly expressed (39,439 TPM) (Fig. 1g; Table S1).

**Table 1:** Venom components of *T. rufonigra*

	Primary structure	TPM	Features
Tr1a	GWKSGAKDCAKKGAECVLQCVKQKM^a^	345,330	Homodimer
Tr1b^b^	GWKSAKECAKKGAECVLQCVKQKM^a^	197,554	
Tr2a	KKKKKKKWIITAFKEAGKIVGEALVGEAISAAVSAATERKE	89,707	GalNAc
Tr2b	KKKKKKKWIITAIKEAGKIVGEALVGEAISAAVSAATEKKE	10,031	GalNAc
Tr3a	FLGAIKALFKAGKIIPTIAELVSRHQQKSQ	65,399	
Tr4a/b	IINMALFKAIANCRDEGKTFQECFKLAEESKRKILKNIKY	1609^c^	
Tr5a	IFGTVLKKITDMFVKCKVKSWIE	254	

*TPM* Transcripts per million

^a^ *C*-Terminal amidation

^b^ No mature peptide detected in the venom

^c^ The TPM value is the sum of more than one transcript

**Fig. 2:**
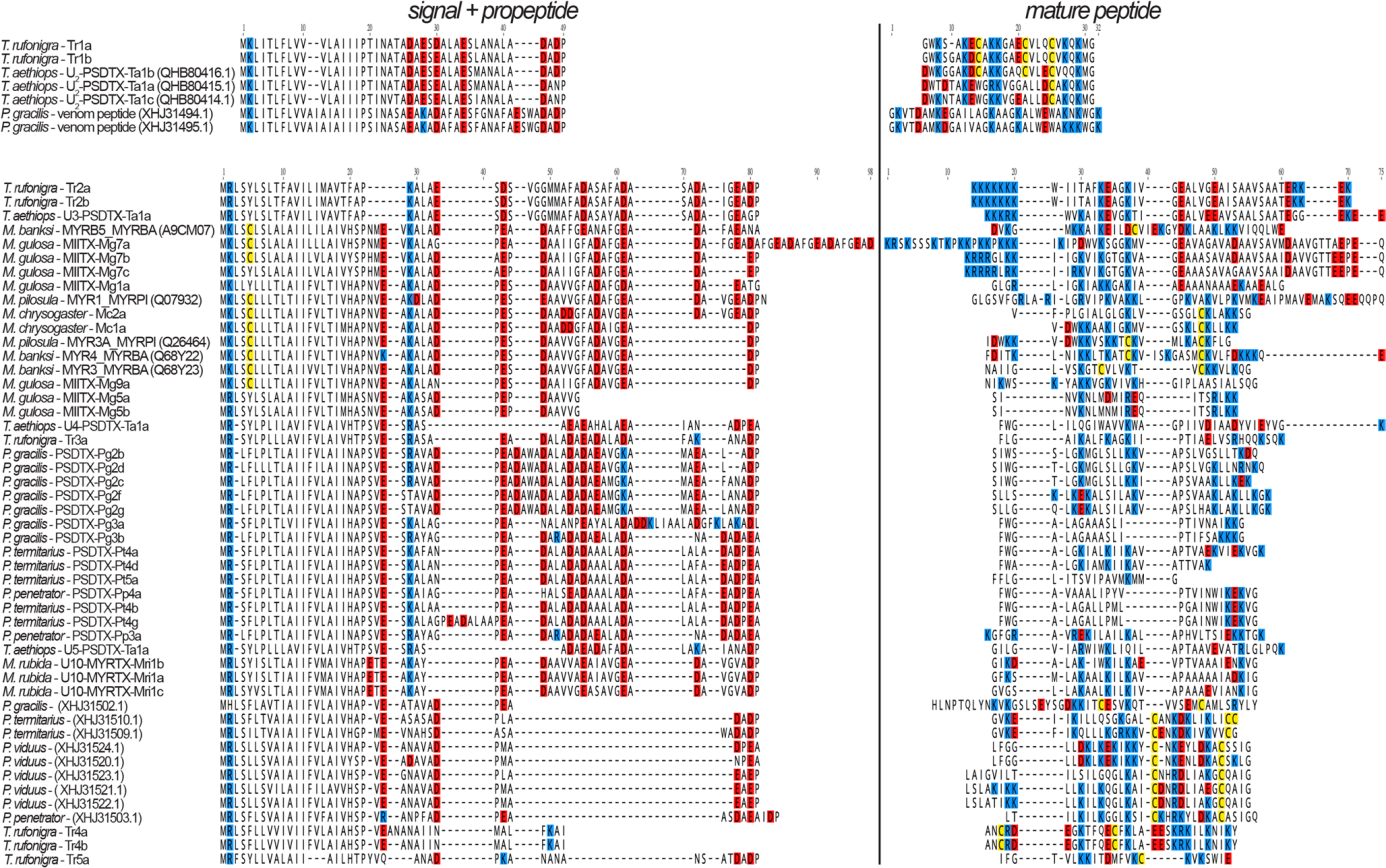
*T. rufonigra* venom peptides are derived from one to two hyperdiverse gene families. Amino acid sequence alignments of the *T. rufonigra* venom peptide precursors. Signal+propeptide and mature peptides were aligned separately. Lysine/arginine, aspartate/glutamate, and cysteine residues are highlighted in blue, red, and yellow, respectively. Post-translational modifications are not shown

Each of the eight *T. rufonigra* venom peptides shared a similar precursor structure and exhibited general similarity in sequence (Fig. 2). We used the BLASTp algorithm to search the UniProt/GenBank protein database for sequences related to the *T. rufonigra* venom peptides (using the precursor sequences of these peptides as queries). The most closely related sequence to Tr1a (90% identity), was U_2_-PSDTX-Ta1b, previously reported from a venom gland transcriptome of *Tetraponera aethiops* (Fig. 2). Like Tr1a, Ta1b has three cysteine residues and was detected as a homodimer in the venom of *T. aethiops* [9]. An additional two peptides from *T. aethiops* share high sequence similarity to Tr1a but have only one cysteine residue, while two sequences from *Pseudomyrmex gracilis* (XHJ31494 and XHJ31495), have a similar signal peptide sequence but distinctive, cysteine-free, mature peptides, the activity of which remain undetermined (Fig. 2). Tr2a had highest sequence similarity (81%) to U_3_-PSDTX-Ta1a from *T. aethiops*, and was also similar to MIITX-Mg7a-c previously reported from the venom of *Myrmecia gulosa* (subfamily Myrmeciinae) (Fig. 2). Mg7a, which like Tr2a, is also a glycopeptide, is cytolytic and causes nociception in mammals and paralysis in insects [28]. For Tr3a, Tr4a and Tr5a, multiple similar sequences were detected from other pseudomyrmecine and myrmeciine ants (Fig. 2).

**Fig. 3:**
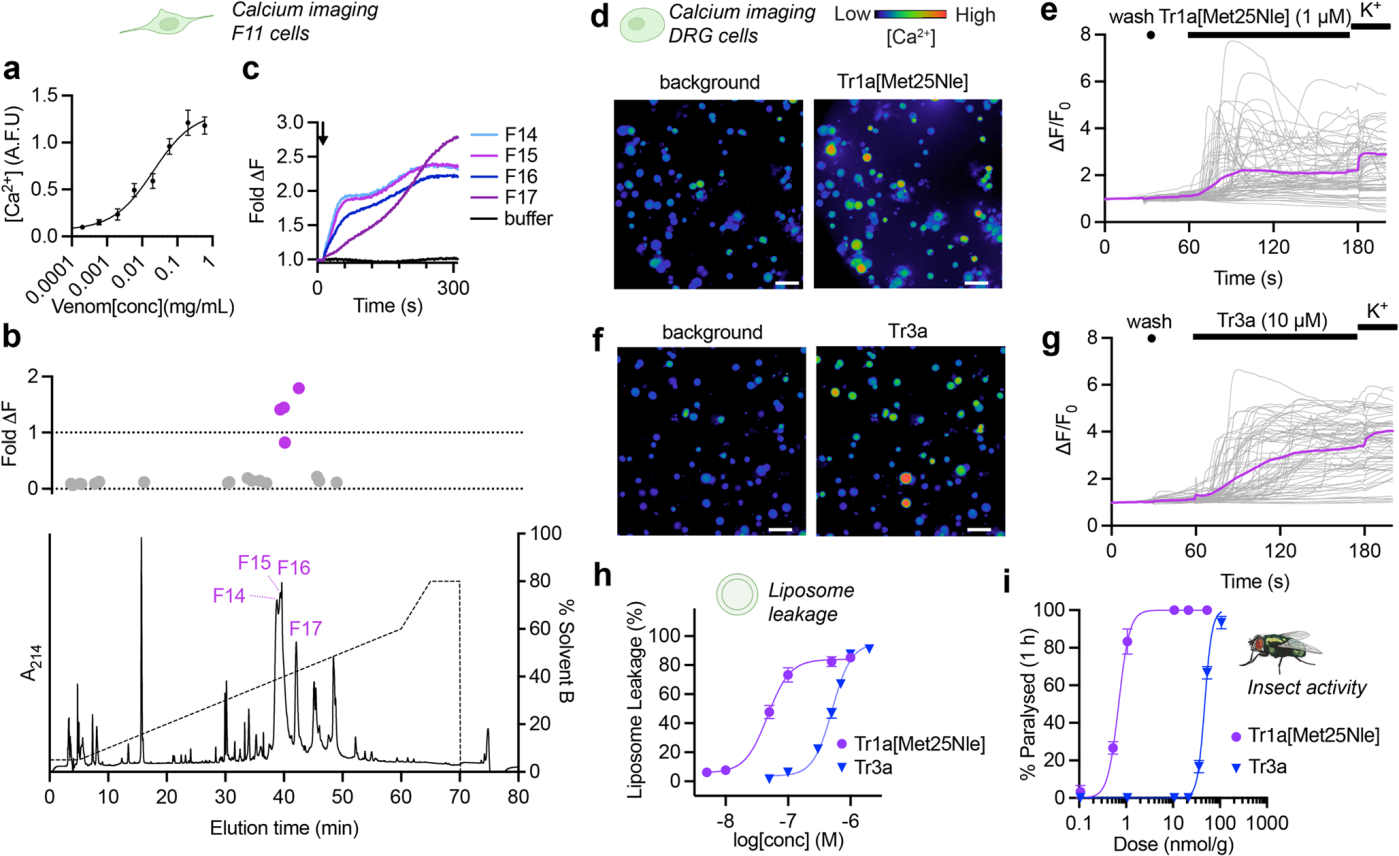
Activity of *T. rufonigra* venom peptides Tr1a and Tr3a. **A** Concentration-dependent effect of *T. rufonigra* venom on F11 cells; EC_50_ = 0.024 ± 0.006 µg/mL. **B** Chromatogram showing fractionation of *T. rufonigra* venom using RP-HPLC and activity of fractions on F11 cells (data are the mean of two replicates); active fractions are colored purple. **C** [Ca^2+^]_*i*_ in F11 cells after addition of f14, f15, f16, and f17. The arrow indicates the time of addition. **D**–**G** Change in [Ca^2+^]_*i*_ in mouse primary DRG cells treated with Tr1a[Met25Nle] (1 µM), and Tr3a (10 µM). Scale bar = 100 μm. **H** Concentration-dependent effect of Tr1a[Met25Nle] and Tr3a on POPC: POPE (1:1) liposomes; EC_50_ = 47 ± 1 nM and 512 ± 21 nM, respectively. **I** Dose-dependent effect of Tr1a[Met25Nle] and Tr3a injection in blowflies; PD_50_[1 h] = 0.70 ± 0.02 nmol/g and 47.4 ± 0.7 nmol/g, respectively; data in panels A, H and I are expressed as mean ± SEM and fitted with a nonlinear regression with variable slope (four parameters)

Envenomation by *T. rufonigra* is painful (personal observation S.N., S.R.) and we were interested in identifying the toxins responsible for this. We first tested our crude venom sample on F11 cells, a hybrid of mouse neuroblastoma and DRG neurons, a model of mammalian nociceptive sensory neurons. Consistent with its pain-causing activity, *T. rufonigra* venom caused a rapid concentration-dependent increase in intracellular Ca^2+^ ([Ca^2+^]_*i*_) in F11 cells with a median effective concentration (EC_50_) of 0.024 ± 0.006 mg/mL (Fig. 3a). Next, we fractionated *T. rufonigra* venom using RP-HPLC and tested fractions for activity on F11 cells. Four fractions (F14–F17) activated F11 cells, causing a rapid increase in intracellular calcium (Fig. 3b, c). Analysis of these fractions by LC-MS/MS revealed that fractions 14 and 15 contained the Tr1a homodimer (theoretical mass = 5382.74 Da, F14 observed mass = 5382.76 Da, F15 observed mass = 5382.74 Da). Fraction 16 contained a derivative of Tr1a (mass 5350.49 Da), with trace amounts of Tr2a and Tr3a also detected. Fraction 17 corresponded to Tr3a (theoretical mass = 3291.92 Da, observed mass = 3291.98 Da), with trace amounts of Tr1a also detected. These data suggested that two *T. rufonigra* venom peptides (Tr1a and Tr3a) are able to activate mammalian sensory neurons, consistent with a role in envenomation-induced pain.

To investigate the mode-of-action of Tr1a and Tr3a, we chemically synthesized the peptides and analysed their activity using high-content calcium imaging of primary cultures of mouse DRG neurons. Synthetic monomeric Tr1a thermodynamically folded and spontaneously formed a homodimer in NH_4_HCO_3_ buffer. The synthetic peptide co-eluted with native Tr1a from *T. rufonigra* venom (fraction 14), indicative of a shared fold (Fig. S3). Tr1a has a single methionine residue (Met25), which we observed was susceptible to oxidation. We therefore synthesised Tr1a[Met25Nle] where Met25 was replaced with the nearly isosteric norleucine. As with Tr1a, synthetic monomeric Tr1a[Met25Nle] thermodynamically folded and spontaneously formed a homodimer in NH_4_HCO_3_ buffer. A comparison of the native and norleucine-containing peptides by 1D ^1^H-NMR spectroscopy was indicative of a shared overall fold (Fig. S4), and the Tr1a[Met25Nle] was therefore used for all subsequent experiments.

Application of Tr1a[Met25Nle] homodimer (1 µM) to DRG cells caused a rapid increase of [Ca^2+^]_*i*_ in both neuronal and non-neuronal cells, followed by leakage of dye into the extracellular medium (Fig. 3d, e). At a concentration of 1 µM, Tr3a had no effect on DRG cells, but at 10 µM caused a gradual sustained increase in [Ca^2+^]_*i*_ in all cells (Fig. 3f, g). The non-specific effects of each peptide were suggestive of a membrane-targeting mode-of-action. Consistent with this activity, both Tr1a[Met25Nle] and Tr3a caused membrane permeabilization in POPC: POPE (1:1) liposomes loaded with fluorescent dye with EC_50_ of 47 ± 1 nM (*n* = 6) and 512 ± 21 nM (*n* = 6), respectively (Fig. 3h).

Many ant venom peptides with cytolytic activity also have insecticidal activity [11, 7, 29, 30, 28]. We tested if Tr1a[Met25Nle] and Tr3a could paralyze adult blowflies (*Lucilia caesar*). Intrathoracic injection of Tr1a[Met25Nle] in blowflies caused a rapidly developing irreversible paralysis (PD_50_[1 h] = 0.70 ± 0.02 nmol/g; Fig. 3i). Tr3a also caused rapid irreversible paralysis, but only at much higher doses (PD_50_[1 h] = 47.4 ± 0.7 nmol/g; Fig. 3i). Together these assays suggest that both Tr1a and Tr3a are membrane-targeting peptides, and that this mechanism of action confers activity on both mammalian sensory neurons and in insects. Of these two peptides, Tr1a was more highly-expressed in the venom gland and was more potent in our assays.

**Fig. 4:**
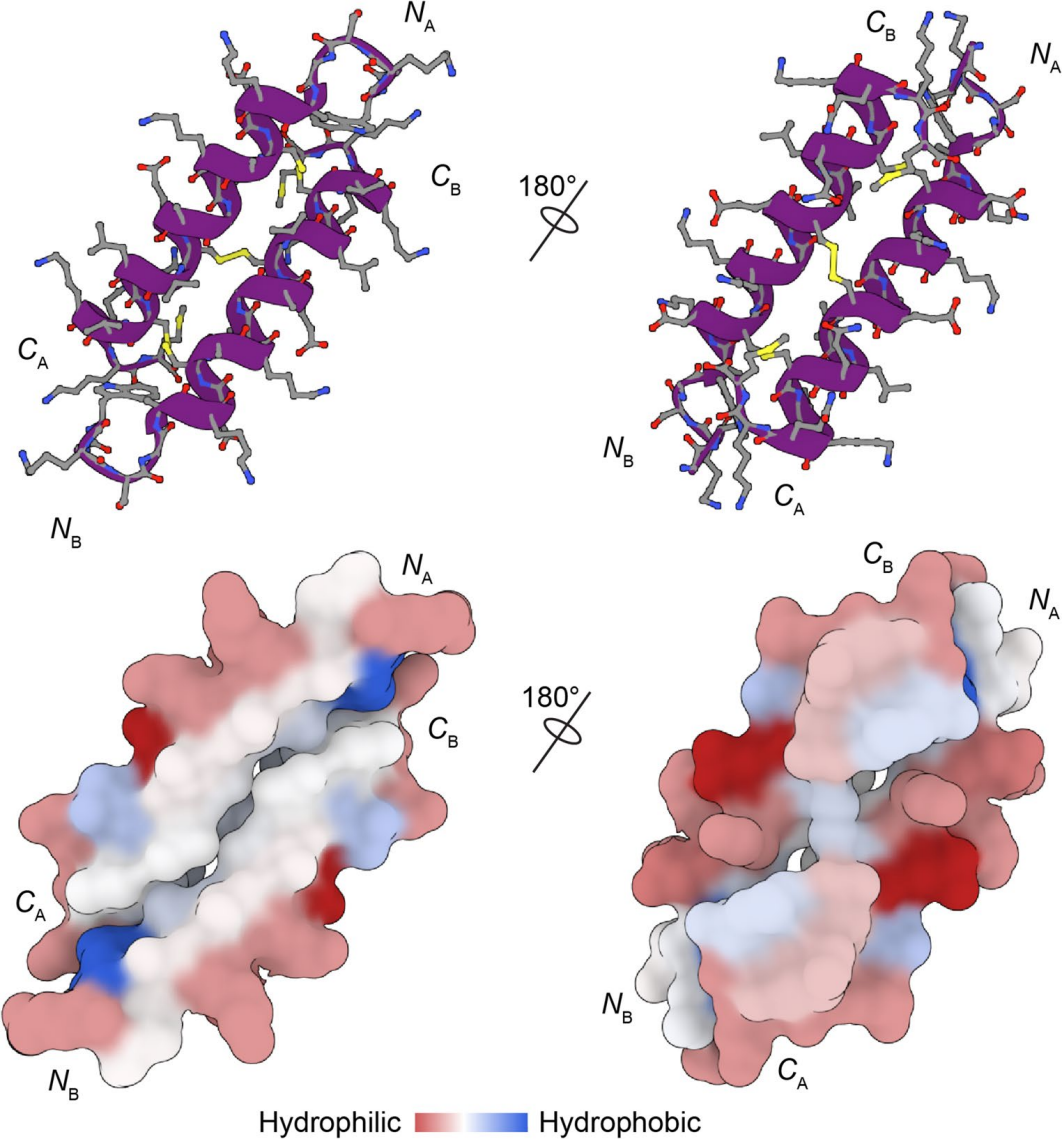
Tr1a is a homodimer of two antiparallel α-helical peptide chains connected by three inter-chain disulfide bonds. Three-dimensional structure of the Tr1a homodimer predicted by AlphaFold3 shown as cartoon with ball and chain representation and molecular surface representation. Molecular surface representation is coloured according to residue hydrophobicity. *N*- and *C*-termini of each chain (A and B) are labelled

Tr1a is a homodimer of two 25 amino acid residue chains. Each chain has three cysteine residues, forming 3 disulfide bonds in the homodimeric peptide. Using AlphaFold3, we predicted the three-dimensional structure of the Tr1a homodimer (Fig. 4; Fig S5). In the predicted three-dimensional structure, the two Tr1a chains are present as α-helices, which are arranged antiparallel to one other and connected by three inter-chain disulfide bonds (Cys9-Cys20, Cys16-Cys16, Cys20-Cys9). The overall structure is symmetrical and characterized by a large polar surface on one face and a large hydrophobic face on the other. Analysis of synthetic Tr1a[Met25Nle] by circular dichroism spectroscopy was consistent with an α-helical structure (Fig. S6), while ^15^N-HSQC NMR spectroscopy indicated a well dispersed set of resonances corresponding to a single polypeptide chain (Fig. S7). These data were consistent with the highly-ordered symmetrical structure predicted by AlphaFold3. We performed structure calculations with Cyana using experimentally-determined distance restraints obtained from 2D NOESY spectra and analysed the structures using Molprobity [19]. Calculations assumed a dimer made up of two covalently linked identical chains of Tr1a[Met25Nle] and disulfide connectivity was restrained to each of the 11 possible isomers. The model with the lowest clashscore, the best Molprobity score and rank and the best stereochemical quality (as exemplified by a high percentage of dihedrals in favoured and additionally allowed regions) corresponded to the disulfide connectivity (1–3’, 2–2’, 3 − 1’) of the predicted Alphafold3 structure (Table S2). Together our data indicate that native Tr1a and synthetic Tr1a and Tr1a[Met25Nle] exist as homodimers with a symmetrical antiparallel configuration of the two peptide chains.

**Fig. 5:**
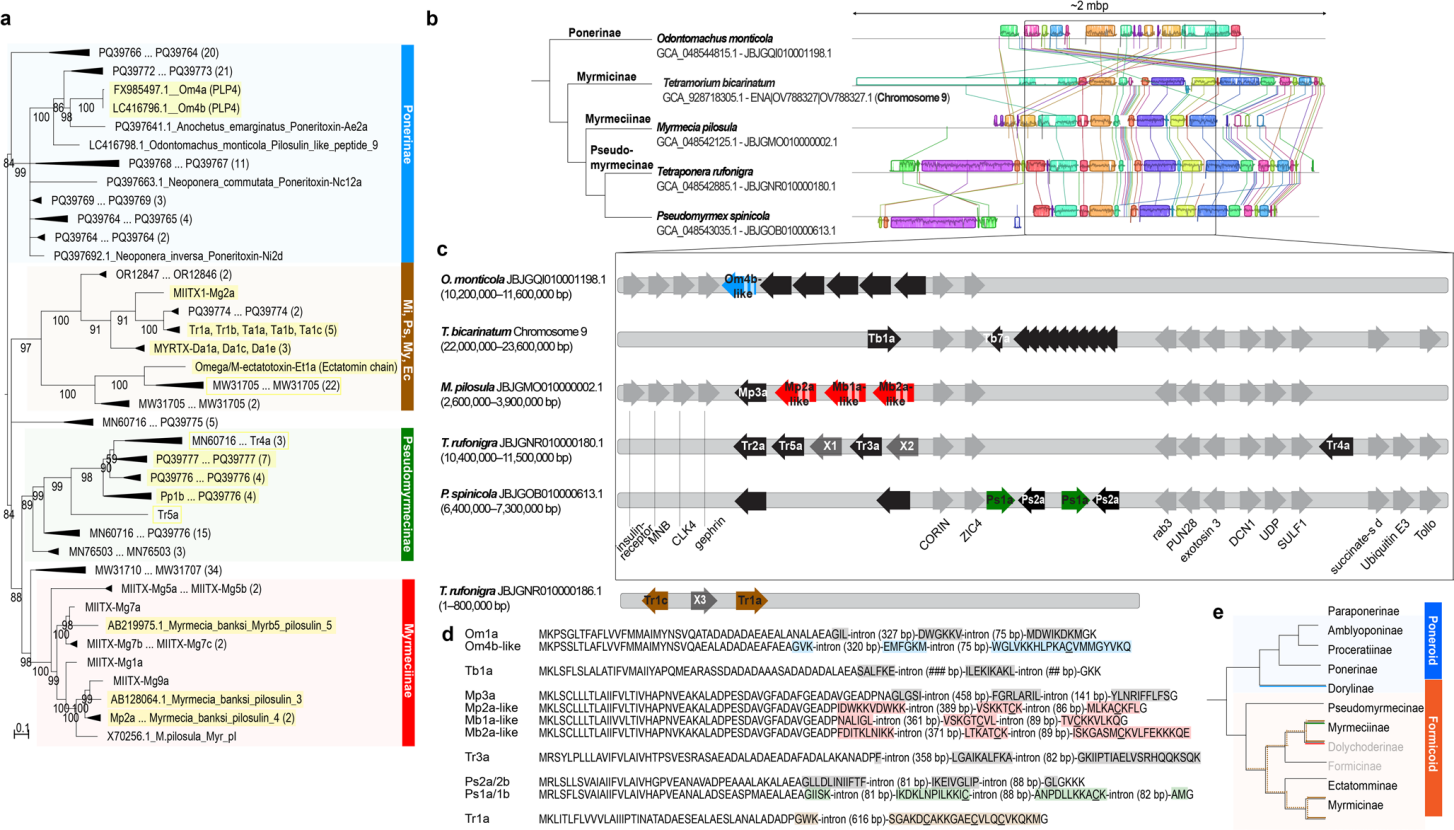
Dimeric ant venom peptides evolved convergently. **A** Phylogenetic tree of ant venom peptide precursors (nucleotide sequence) illustrating multiple clades of dimeric peptides. Number of sequences in each collapsed clade is indicated in parentheses. Precursors of described and putative ant venom dimeric peptides are highlighted and boxed, respectively, in yellow. Mi, Myrmeciinae; Ps, Pseudomyrmecinae; My, Myrmicinae; Ec, Ectatomminae. The sequence alignment is included as File S1. **B** MAUVE alignment of genomic regions containing dimeric venom peptide genes from representative ant species. **C** Local genomic region containing dimeric venom peptide genes. Genes encoding dimeric and non-dimeric venom peptides are shown in colour (according to clade) and black, respectively. Non-venom genes are grey. **D** Translated open reading frames of the genes encoding dimeric and related non-dimeric ant venom peptides. Known or predicted mature peptide sequences are coloured according to clade; Cysteine residues are underlined; For clarity, intron sequences are truncated with sizes indicated. Corresponding nucleotide sequence are included as Fig. S8. **E** Cladogram of selected ant subfamilies (based on [32]) showing distribution of different dimeric ant venom peptide lineages. Coloured branches indicate putative evolutionary origins of each clade of dimeric ant venom peptides. Non-stinging subfamilies, Formicinae and Dolichoderinae, are colored gray

Several other dimeric peptides have been reported in ant venoms (Table 2). However, despite sharing dimeric structures and cytolytic activity, there is limited sequence similarity between many of them (Table 2). We hypothesised that dimeric peptides in ant venoms evolved convergently. To test this, we generated a phylogenetic tree based on an alignment of the precursor sequences (nucleotide; File S1) of described dimeric ant venom peptides and related ant venom peptides (Fig. 5a). The precursor sequences of Tr1a and Tr1b group together with those of Ta1a/b/c from *T. aethiops* (Pseudomyrmecinae), Mg2a from *Myrmecia gulosa* (Myrmeciinae), Da1a/c from *Daceton armigerum* (Myrmicinae) and Ectatomin from *Ectatomma tuberculatum* (Ectatomminae). Precursor sequences of Pp1b from *Pseudomyrmex penetrator*, Pt1 large and small subunits from *Pseudomyrmex triplarinus* as well as several homologues from other Pseudomyrmecine species, form an independent *Pseudomyrmex*-specific clade distinct from the *Tetraponera* dimeric venom peptides. Precursor sequences of Mp2a (*M. pilosula*), Pilosulin-3, Pilosulin-4 (*M. banksii*) and Pilosulin 5 (*M. pilosula*) are part of a Myrmeciinae-specific clade. Finally, precursor sequences of PLP4 (Om4a/b) from *O. monticola* form an independent Ponerinae-specific clade. These data suggested that dimerism of venom peptides evolved on at least four independent occasions in ants.

**Table 2:** Dimeric ant venom peptides

Peptide	Species	Primary structure	
Tr1a	*Tetraponera rufonigra*	GWKSGAKDCAKKGAECVLQCVKQKM^a^	This study
Ta1b	*Tetraponera aethiops*	DWKGGAKDCAKKGAQCVLECVQQKM^a^	[9]
Ta1a	*Tetraponera aethiops*	DWKNTAKEWGKKVGEALLDCAKQKM^a^	[9]
Ta1c	*Tetraponera aethiops*	DWTDTAKEWGRKVGGALLDCAKQKM^a^	[9]
Ectatomin	*Ectatomma tuberculatum*	GVIPKKIWETVCPTVEPWAKKCSGDIATYIKRECGKL	[33, 34]
		WSTIVKLTICPTLKSMAKKCEGSIATMIKKKCDK	[33, 34]
Pilosulin 3 (Mp2a/b)	*Myrmecia pilosula*	IDWKKVDWKKVSKKTCKVMLKACKFL^a^	[35]
		LIGLVSKGTCVLVKTVCKKVLKQ^a^	[35]
Pilosulin 5	*Myrmecia pilosula*	DVKGMKKAIKEILDCVIEKGYDKLAAKLKKVIQQLWE	[36]
Mg2a	*Myrmecia gulosa*	LLSKDQALKHVWGVLKKLGKAAMEYVIQQICAKYNKK	[11]
PLP4 (Om4a)	*Odontomachus monticola*	GVKELFGKAWGLVKKHLPKACGLLGYVKQ	[37]
Pt1 LS1	*Pseudomyrmex triplarinus*	LSLGTIKKLLQILAQGLKAICNHRDLIAKGCQA	[38]
Pt1 LS2		ISLAQIKKLLQIIKQGLKAICDNRDLIAKGCQA	[38]
Pt1 SS1		LFGGLLDKLKEKIKKYCNKENLDKACSKL	[38]
Pt1 SS2		LFGGLLDKLREKIKKYCNKENLDKACSKL	[38]
Pt1 SS3		LFGNIIDKLREKIKKYCNKENLDKACSKL	[38]
Δ-PSDTX-Pp1a A	*Pseudomyrmex penetrator*	KIPNILKGGLKSICKHRKYLDKACAAI	[7]
Δ-PSDTX-Pp1a B	*Pseudomyrmex penetrator*	IDPLTILKILKGGLKSICKHRKYLDKACASIGQ	[7]
Da1a	*Daceton armigerum*	GKNKEALKAQLKECVKAVEKYITDKISKKVLQAINALIDKI	[39]
Da1c	*Daceton armigerum*	GKEKEAFKAQLRECVKAGAKYLSHKLSKAMNALIDKI	[39]

^a^ *C*-terminal amidation

To further investigate the evolution of dimeric venom peptides in ants we analysed the genomes of *T. rufonigra* and representatives of each of the other ant lineages. In the genome assembly of *T. rufonigra* (GenBank: GCA_048542885.1), most of the *T. rufonigra* venom peptide genes (Tr2a, Tr3a, Tr4a and Tr5a) are found together on a single contig alongside several related putative pseudogenes (similar signal peptides but lacking a mature peptide region) (Fig. 5b, c). The Tr1a gene, together with a paralog (Tr1c), is located on a different contig. We aligned these regions to the chromosome-level genome assembly of *Tetramorium bicarinatum* (Myrmecinae; GenBank: GCA_928718305.1). The region encoding Tr2a, Tr3a, Tr4a and Tr5a corresponds to the A1 gene superfamily locus of chromosome 9 of *T. bicarinatum* (Fig. 5b, c). We could not identify an orthologous region for Tr1a in the *T. bicarinatum* genome.

In the *T. bicarinatum* genome, the A1 locus contains a cluster of 14 venom peptide-encoding genes, although none are known to be dimeric [31]. Tr2a, Tr3a and Tr5a most closely align with the MYRTX_A1_-Tb1a venom peptide gene of *T. bicarinatum* (UniProt accession: A0A088SG62), with which they share a similar gene structure with three exons and two introns located within the mature peptide-encoding sequence (Fig. 5d; Fig. S8). This is distinct from the gene structure of Tr1a, which consists of two exons with the single intron located within the mature peptide-encoding sequence (Fig. 5d; Fig. S8).

Next, we investigated the genes encoding dimeric venom peptides in the genomes of representatives of the Ponerinae (*O. monticola*; GCA_048544815.1), Myrmeciinae (*M. pilosula*; Genbank: GCA_048542125.1) and Pseudomyrmex (*Pseudomyrmex spinicola*; Genbank: GCA_048543035.1). We identified dimeric venom peptide genes in the genomes of each of these species, including a single Om4b-like gene for *O. monticola*, Mp2a-, Mb1a and Mb2a-like genes for *M. pilosula*, and two genes for *P. spinicola* (encoding Pp1a-like dimeric peptides we have called Ps1a and Ps1b). Regardless of lineage, all of these genes are located near the Tetramorium A1 locus (Fig. 5b, c) and in tandem with genes encoding non-dimeric venom peptides (Fig. 5c). Those of *O. monticola* and *M. pilosula* align most closely with the region containing the MYRTX_A1_-Tb1a gene of *T. bicarinatum* (and Tr2a, Tr3a and Tr5a of *T. rufonigra*), with which they share a similar gene structure (three exons and two introns located within the mature peptide-encoding sequence). The dimeric venom peptide genes of *P. spinicola* align with a distinct region of the A1 locus, and have a distinct gene structure consisting of four exons with each intron located within the mature peptide-encoding sequence.

Together these data suggest that the clade of dimeric ant venom peptides to which Tr1a belongs either evolved in ancestral formicoids or following the divergence of formicoids, evolved in parallel in different formicoid ant lineages from the same gene family (Fig. 5e). The dimeric venom peptides of *Pesudomyrmex* evolved independently of those of *Tetraponera* and other ants. The genes encoding dimeric venom peptides of the Ponerinae and Myrmeciinae, while located in a similar genomic location, are nested within independent clades of non-dimeric venom peptide genes (Fig. 5a, c; Fig. S8). On the basis of these data, the most-parsimonious explanation is that the genes encoding the dimeric venom peptides of the Ponerinae and Myrmeciinae likely share deep homology, but that dimerism evolved independently in each.

## Discussion

Animal venoms are renowned as a source of diverse structurally-constrained disulfide-bonded peptides. For example, the cysteine-rich venom peptides of scorpions, spiders and cone snails typically adopt well-defined folds with intra-chain disulfide bonds, such as the inhibitor cystine knot (ICK), kunitz or defensin folds [40–42]. In most cases these are thought to have inherited and retained their disulfide-bonded structural folds from the endogenous peptides/proteins from which they were recruited, followed by diversification and neofunctionalization [41, 42].

By contrast, the majority of described ant venom peptides are cysteine-free. Most of them adopt amphipathic α-helical structures in membrane-mimicking solvents and are cytolytic [1, 3, 11, 43]. Where cysteine residues do occur in ant venom peptides, such as with Tr1a, they form inter-chain disulfide bonded dimers rather than internally cross-linked monomers (although for exceptions see: [44, 45]). These dimeric peptides also have amphipathic α-helical structures and cytolytic activity. In this study, we demonstrate that several distinct clades of dimeric ant venom peptides exist, including homodimers formed by identical peptide chains linked by one or more inter-chain disulfide bonds, as well as heterodimers composed of two distinct peptide chains (Fig. 4; Table 2).

Our phylogenetic and sequence analyses indicate that these dimeric peptide toxins likely evolved multiple times from ancestral monomeric, cysteine-free venom peptides. The repeated evolution of dimeric peptides from non-dimeric peptides is suggestive of adaptation. Previous functional studies of dimeric ant venom peptides have indicated that dimerization can confer increased potency and stability. For example, Mp2a/b (M-myrmeciitoxin-Mp2a/b) from the venom of *Myrmecia pilosula* (Myrmeciinae) is a heterodimer [35] with potent cytolytic activity on mammalian neuronal and red blood cells, causes nociception and is insecticidal, yet, each individual chain alone is inactive [10, 29]. Similarly, individual chains of Δ-PSDTX-Pp1a A/B from the venom of *Pseudomyrmex penetrator* have markedly reduced insecticidal activity compared with the heterodimer [7]. Moreover, the individual chains of Δ-PSDTX-Pp1a are more rapidly digested by proteinase K (half-life < 10 min) than the venom heterodimer (half-life ~ 13 h) [7]. The increased potency and stability of these dimeric peptides is likely associated with the stabilization of the amphipathic α-helical secondary structure conferred by the disulfide bonds [10, 7].

Together these data allow us to propose a model of the evolution of dimeric ant venom peptides. In this model, a mutation results in a codon for a cysteine residue in a previously monomeric venom peptide sequence, enabling the formation, in the venom, of a disulfide-linked homodimeric peptide. If the resulting dimer has advantageous properties – such as increased potency or stability – selection will favour retention and fixation of this variant in the population. Subsequent mutations introducing additional cysteine residues that further improve the peptide’s stability or potency would result in homodimers with multiple disulfide bonds e.g. Tr1a. Allelic variation, gene duplication followed by sequence divergence, or independent mutation of a second peptide gene could give rise to two distinct cysteine-containing peptide chains in a venom. If the heterodimeric complex forms and confers greater fitness compared with the homodimers, then selection would favour the retention and maintenance of the heterodimeric venom peptide.

Despite these insights, our conclusions remain limited by the currently available data. To date, only a small number of dimeric ant venom peptides have been identified (Table 2), and even fewer have been functionally characterized. Broader sampling to identify additional dimeric ant venom peptides and their monomeric relatives, will allow a better resolved reconstruction of the evolution of these peptides. Nevertheless, this study highlights the malleability of ant venom peptide genes and the role of dimerization in these peptides to confer improved function.

## Supplementary Information

Below is the link to the electronic supplementary material.

Supplementary Material 1

Supplementary Material 2

## Data Availability

Prepropeptide sequences of PSDTX_1_-Tr1a, PSDTX_1_-Tr1b, PSDTX_1_-Tr2a, PSDTX_1_-Tr2b, PSDTX_1_-Tr3a, PSDTX_1_-Tr4a, PSDTX_1_-Tr4b and PSDTX_1_-Tr5a have been deposited with GenBank, under accessions: PX564871, PX564872, PX564873, PX564874, PX564875, PX564876, PX564877, and PX564878, respectively. *T. rufonigra* RNA-seq reads have been deposited in the NCBI sequence read archive under accession SRR37381468. Mass spectrometry data have been deposited to the ProteomeXchange Consortium via the PRIDE partner repository with the dataset identifier PXD078479.
