# Supplementary material for: Venom composition of the ant Tetraponera rufonigra reveals insights into the evolution of dimeric ant venom peptides
Source: Cell Mol Life Sci. 2026 Jun 12;83(1):311. doi: 10.1007/s00018-026-06293-z (PMC13486460; doi:10.1007/s00018-026-06293-z)
Supplement: Supplementary file 1 [file 18_2026_6293_MOESM1_ESM.docx]

**SUPPLEMENTAL IMFORMATION**

**Venom composition of the ant *Tetraponera rufonigra* reveals insights into the evolution of dimeric ant venom peptides**

Suwatjanee Naephrai,^1,2,3^ Hue N. T. Tran,^3^ Thomas Durek,^3,4^ Axel Touchard,^5^ Vanessa Schendel,^3^ William Atherton,^3^ Irina Vetter,^3,6^ Panchika Prangkio,^1,7^ Samuel D. Robinson^3*^

^1^Department of Chemistry, Faculty of Science, Chiang Mai University, Chiang Mai 50200, Thailand.

^2^Graduate Program in Biotechnology, Multidisciplinary and Interdisciplinary School, Chiang Mai University, Chiang Mai 50200, Thailand.

^3^Institute for Molecular Bioscience, The University of Queensland; QLD 4072, Australia.

^4^Australian Research Council Centre of Excellence for Innovations in Peptide and Protein Science, The University of Queensland, Brisbane, QLD 4072, Australia.

^5^CNRS, UMR Ecologie des forêts de Guyane – EcoFoG, Campus Agronomique, BP 316, 97379 Kourou Cedex, France.

^6^School of Pharmacy and Pharmaceutical Sciences, The University of Queensland; Brisbane, QLD 4102, Australia.

^7^Center of Excellence in Material Science and Technology, Faculty of Science, Chiang Mai University, Chiang Mai 50200, Thailand.

*Corresponding author. Email: sam.robinson@uq.edu.au.

**Figure S1. Tr1a is present in the native venom as a homodimer.** A) Total ion chromatogram from LC-MS/MS analysis of native *T. rufonigra* venom with peaks corresponding to the Tr1a homodimer and derivatives thereof labelled. B) Precursor ions derived from the peak at 10.3 min. Charge series of +6 to +9 indicate a peptide with molecular mass of 5382.74 Da (theoretical homodimer mass = 5382.67). MS/MS fragment ions were consistent with the Tr1a sequence.

**Figure S2. Tr3a is a glycopeptide.** A) Precursor ions derived from peak corresponding to Tr3a. Charge series of +5 to +8 indicate a peptide with molecular mass of 4614.70 Da. B) CID-MS/MS (m/z range 100–250) spectrum of the [M+6H]^6+^ precursor ion illustrating oxonium ions.

**Fig. S3. Native and synthetic Tr1a co-elute on HPLC.** HPLC chromatogram (Absorbance at 214 nm) of A) native Tr1a isolated from *T. rufonigra* venom (F14), B) 0.1 nmol oxidised synthetic Tr1a and C) native Tr1a isolated from *T. rufonigra* venom (F14) and 0.1 nmol oxidised synthetic Tr1a. Flow rate 0.3 mL/min; gradient of 0–60% solvent B over 30 min (A: 0.05% TFA, B: 90% ACN, 0.045% TFA).

**Fig. S4. 1D ^1^H-NMR spectra of Tr1a and Tr1a[Met25Nle] indicate a shared overall structural fold.** 1D ^1^H spectra of Tr1a and Tr1a[Met25Nle]. The Tr1a spectrum was acquired at 298 K on a Bruker Avance III 600 MHz spectrometer, using a 1 mg/mL sample at pH 3.5 in water/D_2_O 9:1 (v/v). The Tr1a[Met25Nle] spectrum were acquired at 298 K on a Bruker Avance III 600 MHz spectrometer, using a 5 mg/mL sample at pH 5.8 in water/D_2_O 9:1 (v/v).

**Fig. S5**. **Three-dimensional structure prediction of Tr1a homodimer by AlphaFold3.**

**Fig. S6**. **Tr1a[Met25Nle] adopts α-helical secondary structure in aqueous buffer.** Far-UV CD spectra (260–185 nm) of Tr1a[Met25Nle] (19 μM) in 10 mM K_2_HPO_4_, pH 7.4.

**Fig. S7. Tr1a[Met25Nle] adopts a single major conformation in solution that is consistent with a symmetrical dimeric structure.** 2D [^1^H-^15^N]-HSQC spectrum of Tr1a[Met25Nle]. Spectra were acquired at 298 K on a Bruker Avance III 600 MHz spectrometer, using a 5 mg/mL sample of Tr1a[Met25Nle] at pH 5.8 in water/D_2_O 9:1 (v/v).

**Fig. S8. Dimeric ant venom peptides have diverse gene structures.** Partial open reading frames of the genes encoding selected dimeric and related non-dimeric ant venom peptides are shown with corresponding translated sequence and intron locations. Known or predicted mature peptide sequences are coloured according to the clades defined in **Fig. 5**; Cysteine residues are in bold; For clarity, intron sequences are truncated.

**Table S1. Proteins detected in reduced, alkylated, trypsin-digested sample of *T. rufonigra* venom (> 60% coverage).**

|  | TPM |
| --- | --- |
| PLA_1_ | 39,439^a^ |
| Lipase | 5,263^a^ |
| Venom allergen 3 | 3,678^a^ |
| 5NUC | 1,240 |
| PLA_2_ | 729 |
| DPP-4 | 674 |

TPM, transcripts per million; ^a^, The TPM value is the sum of more than one transcript; PLA_1_, phospholipase-A_1_; 5NUC, 5'-nucleotidase; PLA_2_, phospholipase-A_2_; DPP-4, dipeptidyl peptidase 4.

**Table S2: Evaluation of disulfide connectivity for Tr1a[Met25Nle] using NMR-derived distance and symmetry restraints**

| Connectivity | Schematic | Clashscore^a^ | Molprobity score  (rank)^a^ | Violations^b^ | Target function^b^ | Ramachandran  Favoured (%)^a^ | Ramachandran Allowed (%)^a^ |
| --- | --- | --- | --- | --- | --- | --- | --- |
| 1-3’, 2-2’, 3-1’ |  | 0.33 | 2.25 (61.5%) | 2 | 0.31 | 82.3 | 15.5 |
| 1-2, 3-3’, 1’-2’ |  | 0.73 | 2.51 (47.4%) | 0 | 0.23 | 76.4 | 20.7 |
| 1-2, 3-1’, 2’-3’ |  | 0.95 | 2.48 (48.7%) | 16 | 0.85 | 80.6 | 15.3 |
| 1-1’, 2-2’, 3-3’ |  | 1.25 | 2.55 (45.4%) | 26 | 0.67 | 74.1 | 24.1 |
| 1-3, 2-2’, 1’-3’ |  | 1.52 | 2.67 (39.4%) | 2 | 0.63 | 64.3 | 32.3 |
| 1-1’, 2-3, 2’-3’ |  | 1.91 | 2.67 (39.9%) | 13 | 1.28 | 66.8 | 30 |
| 1-1’, 2-3’, 3-2’ |  | 2.05 | 2.68 (39.1%) | 1 | 0.32 | 71.1 | 25 |
| 1-3, 2-3’, 1’-2’ |  | 2.24 | 2.66 (40.4%) | 45 | 0.43 | 79.3 | 16.8 |
| 1-2’, 2-3, 1’-3’ |  | 2.77 | 2.91 (28.1%) | 127 | 2.31 | 65.2 | 32.9 |
| 1-2’, 2-1’, 3-3’ |  | 2.9 | 2.94 (26.2%) | 18 | 1.2 | 70.9 | 27.3 |
| 1-3’, 2-1’, 3-2’ |  | 3.63 | 2.94 (27.4%) | 40 | 0.79 | 64.1 | 33.2 |

Data are sorted based on Molprobity clashscore from lowest (best) to highest (worst). ^a^: from Molprobity, ^b^: from CYANA.
